# Supplementary material for: Self-stigma and cognitive fusion in parents of children with autism spectrum disorder. The moderating role of self-compassion
Source: PeerJ. 2021 Dec 16;9:e12591. doi: 10.7717/peerj.12591 (PMC8684717; doi:10.7717/peerj.12591)
Supplement: Supplemental Information 2 [file peerj-09-12591-s002.docx]

**Skala postrzeganej stygmatyzacji społecznej**

Perceived Public Stigma Scale, PPSS; Chan & Lam, 2017

polskie tłumaczenie: Pyszkowska & Rożnawski, 2020

Poniżej znajdują się stwierdzenia dotyczącego tego, w jaki sposób osoby z zaburzeniami ze spektrum autyzmu mogą być postrzegane przez otaczających je ludzi. Zastanów się i zaznacz, na ile zgadzasz się z tymi stwierdzeniami. Jeśli nie jesteś pewien/pewna którejś odpowiedzi, proszę, wpisz najbardziej pasującą. To ważne, aby wypełnić wszystkie pytania. Nie ma złych, ani dobrych odpowiedzi, nie ma też podchwytliwych pytań.

**Skala odpowiedzi:**

0 – zdecydowanie się nie zgadzam;

1 – nie zgadzam się;

2 – trochę się nie zgadzam;

3 – trochę się zgadzam;

4 – zgadzam się;

5 – zdecydowanie się zgadzam

|  | 0 – zdecydowanie się nie zgadzam | 1 –  nie zgadzam się | 2 –  trochę się nie zgadzam | 3 –  trochę się zgadzam | 4 – zgadzam się | 5 – zdecydowanie się zgadzam |
| --- | --- | --- | --- | --- | --- | --- |
| 1. Większość ludzi uważa, że autyzm to oznaka osobistej porażki. | 0 | 1 | 2 | 3 | 4 | 5 |
| 2. Większość ludzi uważa osoby z autyzmem za gorsze od innych. | 0 | 1 | 2 | 3 | 4 | 5 |
| 3. Większość ludzi w moim otoczeniu czuje się niespokojnie i/lub niekomfortowo podczas spotkania z osobą z autyzmem. | 0 | 1 | 2 | 3 | 4 | 5 |
| 4. Większość osób w moim otoczeniu czuje smutek, gdy spotyka kogoś z autyzmem. | 0 | 1 | 2 | 3 | 4 | 5 |
| 5. Większość osób w moim otoczeniu traktowałaby osobę z autyzmem dokładnie tak samo, jak innych ludzi. | 0 | 1 | 2 | 3 | 4 | 5 |
| 6. Większość ludzi chętnie zaakceptowałaby osobę z autyzmem jako bliskiego przyjaciela. | 0 | 1 | 2 | 3 | 4 | 5 |
| 7. Większość pracodawców zatrudni osobę z autyzmem, jeśli on/ona ma odpowiednie kwalifikacje. | 0 | 1 | 2 | 3 | 4 | 5 |
| 8. Większość młodych mężczyzn i kobiet byłaby niechętna, aby umówić się na randkę z osobą z autyzmem. | 0 | 1 | 2 | 3 | 4 | 5 |
