# Supplementary material for: Self-stigma and cognitive fusion in parents of children with autism spectrum disorder. The moderating role of self-compassion
Source: PeerJ. 2021 Dec 16;9:e12591. doi: 10.7717/peerj.12591 (PMC8684717; doi:10.7717/peerj.12591)
Supplement: Supplemental Information 3 [file peerj-09-12591-s003.docx]

**Skala postrzeganej stygmatyzacji osób bliskich**

Courtesty Stigma Scale; Chan & Lam, 2017

polskie tłumaczenie: Pyszkowska & Rożnawski, 2020

Poniżej znajdują się stwierdzenia dotyczącego tego, w jaki sposób osoby z zaburzeniami ze spektrum autyzmu oraz ich bliscy mogą być postrzegane przez otaczających je ludzi. Zastanów się i zaznacz, na ile zgadzasz się z tymi stwierdzeniami. Jeśli nie jesteś pewien/pewna którejś odpowiedzi, proszę, wpisz najbardziej pasującą. To ważne, aby wypełnić wszystkie pytania. Nie ma złych, ani dobrych odpowiedzi, nie ma też podchwytliwych pytań.

**Skala odpowiedzi:**

0 – zdecydowanie się nie zgadzam;

1 – nie zgadzam się;

2 – trochę się nie zgadzam;

3 – trochę się zgadzam;

4 – zgadzam się;

5 – zdecydowanie się zgadzam

|  | 0 – zdecydowanie się nie zgadzam | 1 –  nie zgadzam się | 2 –  trochę się nie zgadzam | 3 –  trochę się zgadzam | 4 – zgadzam się | 5 – zdecydowanie się zgadzam |
| --- | --- | --- | --- | --- | --- | --- |
| 1. Większość osób obwinia rodziców za to, że ich dzieci mają autyzm. | 0 | 1 | 2 | 3 | 4 | 5 |
| 2. Większość ludzi uważa, że rodzice dzieci z autyzmem nie są tak odpowiedzialni i troskliwi, jak inni rodzice. | 0 | 1 | 2 | 3 | 4 | 5 |
| 3. Większość ludzi nie traktowałaby rodzin, których członek ma autyzm, w taki sam sposób, w jaki traktują inne rodziny. | 0 | 1 | 2 | 3 | 4 | 5 |
| 4. Większość ludzi patrzy z góry na rodziny, w których jest osoba z autyzmem. | 0 | 1 | 2 | 3 | 4 | 5 |
| 5. Większość osób w mojej społeczności wolałaby nie przyjaźnić się z rodzinami, w których jest osoba z autyzmem. | 0 | 1 | 2 | 3 | 4 | 5 |
| 6. Większość osób wolałaby nie odwiedzać rodzin, w których jest osoba z autyzmem. | 0 | 1 | 2 | 3 | 4 | 5 |
| 7. Większość osób uważa, że ich przyjaciele nie odwiedzaliby ich tak często, jeśli członek ich rodziny był w terapii z powodu autyzmu. | 0 | 1 | 2 | 3 | 4 | 5 |
