# Supplementary material for: Self-stigma and cognitive fusion in parents of children with autism spectrum disorder. The moderating role of self-compassion
Source: PeerJ. 2021 Dec 16;9:e12591. doi: 10.7717/peerj.12591 (PMC8684717; doi:10.7717/peerj.12591)
Supplement: Supplemental Information 4 [file peerj-09-12591-s004.docx]

**Krótka skala współczującej postawy wobec samego siebie**

(Raes, Pommier, Neff, Van Gucht, 2011)

**Instrukcja:** Proszę, odpowiedz na wszystkie pytania najlepiej, jak potrafisz. Jeśli nie jesteś pewien/pewna którejś odpowiedzi, proszę, wpisz najbardziej pasującą. To ważne, aby wypełnić wszystkie pytania. Nie ma złych, ani dobrych odpowiedzi, nie ma też podchwytliwych pytań.

| **1** | **2** | **3** | **4** | **5** |
| --- | --- | --- | --- | --- |
| zupełnie do mnie nie pasuje | słabo do mnie pasuje | trochę do mnie pasuje | raczej do mnie pasuje | bardzo do mnie pasuje |

| ..…. | 1.Kiedy nie podołam ważnej dla mnie rzeczy, przepełnia mnie poczucie własnej nieudolności. |
| --- | --- |
| ..…. | 2.Staram się wykazywać cierpliwość i zrozumienie wobec elementów mojej osobowości, których nie lubię. |
| ..…. | 3.Kiedy wydarza się coś bolesnego, staram się zachować wyważone spojrzenie na sytuacje |
| ..…. | 4.Kiedy jestem przygnębiony, mam tendencję do postrzegania innych ludzi jako prawdopodobnie szczęśliwszych ode mnie. |
| ..…. | 5.Staram się postrzegać porażki jako część ludzkiej natury. |
| ..…. | 6.Kiedy przechodzę przez bardzo trudny czas, okazuję sobie troskę i czułość, której potrzebuję |
| ..…. | 7.Kiedy coś mnie zdenerwuje, staram się zachować równowagę emocjonalną. |
| ..…. | 8.Kiedy nie powodzi mi się coś ważnego, czuję się samotny we własnej porażce. |
| ..…. | 9.Kiedy jestem przygnębiony, mam obsesję na punkcie wszystkiego co jest źle. |
| ..…. | 10.Kiedy czuję się w jakiś sposób nieudolny, staram się sobie przypomnieć, że to odczucie podziela większość ludzi. |
| ..…. | 11.Jestem krytyczny i potępiam moje wady i niedociągnięcia. |
| ..…. | 12.Jestem nietolerancyjny i niecierpliwy wobec pewnych elementów mojej osobowości. |

**Klucz**

**1. Życzliwość dla Siebie (ŻS).** Auto-życzliwość- zdolność do traktowania siebie raczej z troską niż z ostrym osądem. (α Cronbacha = 0,55) **(2, 6)**

**2. Osądzanie Siebie (OS).**  (α Cronbacha = 0,81) **(11, 12)**

**3.Wspólne Doświadczenie (WD).** Zwyczajne człowieczeństwo- postrzeganie braku perfekcji bardziej jako wspólnego elementu ludzkiego doświadczenie niż uczucia, że jest ono wyjątkowe dla jednostki i ponoszonych przez nią porażek. Poczucie wspólnoty doświadczeń z całym gatunkiem ludzkim(α Cronbacha = 0,60) **(5, 10)**

**4.Izolacja (I). I – Isolation.** Poczucie odizolowania od świata w kontekście swoich doświadczeń (α Cronbacha = 0,77) **(4, 8)**

**5.Refleksyjna Świadomość (RŚ) –** Mindfulness, Uważność na własne odczucia- bardziej zrównoważone podejście do własnych doświadczeń niż wyolbrzymianie i tworzenie dramatycznej fabuły własnego cierpienia. Cierpliwa świadomość własnych odczuć i doznań, bez próby ich krytykowania, kontrolowania, tłumienia i wypierania. Jest to refleksyjne spostrzeganie swoich odczuć, dzięki czemu człowiek może zyskać dystans wobec nich. Refleksyjnej świadomości własnych doświadczeń (α Cronbacha = 0,64) **(3, 7)**

**6. Nadientyfikacja (N).** Przesadne identyfikowanie się z własnymi przeżyciami, zwłaszcza negatywnymi, czyli z własnymi wadami, słabościami i błędami (α Cronbacha = 0,69) **(1, 9)**

**Krótki opis narzędzia**

xxx

**Bibliografia**

1. Dzwonkowska, I. (2011). Współczucie wobec samego siebie (self-compassion) jako moderator wpływu samooceny globalnej na afektywne funkcjonowanie ludzi. *Psychologia Społeczna*, (1 (16)), 49-66.
2. Raes, F., Pommier, E., Neff, K. D., & Van Gucht, D. (2011). Construction and factorial validation of a short form of the self‐compassion scale. *Clinical psychology & psychotherapy,* 18(3), 250-255.
3. Neff, K.D. (2003a). Development and validation of a scale to measure self-compassion. *Self and Identity*, 2, 223–250.
4. Neff, K.D. (2003b). Self-compassion: An alternative conceptualization of a healthy attitude toward oneself. *Self and Identity*, 2, 85–102.
5. Neff, K.D. (2008). Self-compassion: Moving beyond the pitfalls of a separate self-concept. In J. Bauer & H.A. Wayments (Eds), Transcending self-interest: Psychological explorations of the quiet ego (pp. 95–106). Washington, DC: APA Books.
6. Neff, K.D. (2009). Self-compassion. In M.R. Leary & R.H. Hoyle (Eds), Handbook of individual differences in social behavior (pp. 561–573). New York: Guilford Press.
7. Neff, K.D., Kirkpatrick, K.L., & Rude, S.S. (2007). Self-compassion and adaptive psychological functioning. *Journal of Research in Personality*, 41, 139–154.
8. Neff, K.D., Rude, S.S., & Kirkpatrick, K.L. (2007). Anexamination of self-compassion in relation to positive psychological functioning and personality traits. *Journal of Research in Personality,* 41, 908–916.
9. Neff, K.D., & Vonk, R. (2009). Self-compassion versus global self-esteem: Two different ways of relating to oneself. *Journal of Personality*, 77, 23–50.
